# Targeted Therapies in Neuroblastoma: A History and a View to the Future

**DOI:** 10.3390/cancers18111742

**Published:** 2026-05-27

**Authors:** Nilay Shah

**Affiliations:** 1 Division of Hematology/Oncology/BMT, Nationwide Children’s Hospital, Columbus, OH 43205, USA; nilay.shah@nationwidechildrens.org; 2 Department of Pediatrics, The Ohio State University College of Medicine, Columbus, OH 43210, USA

**Keywords:** neuroblastoma, targeted therapies, pediatric oncology, MYCN, GD2, differentiation, precision medicine

## Abstract

Neuroblastomas remain a disproportionately morbid and mortal pediatric cancer. Representing 8% of all childhood cancer diagnoses, it still is the cause of 14% of cancer-related deaths in children despite the use of aggressive multimodal treatments with significant long-term sequelae. The development of targeted therapies for neuroblastoma has been and will continue to be the optimal approach to improve durable long-term survival in these patients while reducing the consequences of treatment. Here, I review the history of the development of targeted therapies for the treatment of neuroblastoma. I discuss the history of therapies that have been integrated into current treatment regimens, including isotretinoin, anti-GD2 antibodies and eflornithine. I review the history of candidate biologic targets in this malignancy as well as ongoing and future efforts to improve outcomes.

## Simple Summary

Neuroblastoma is an embryonal tumor of the sympathetic nervous system. While it accounts for 7-8% of all childhood cancers, it disproportionately accounts for 14% of all childhood cancers deaths. Furthermore, survivors are left with significant long-term sequelae from current conventional therapies. Conventional chemotherapy can be helpful in many cases, but it has been the use of targeted therapeutics that has significantly improved outcomes. That said, significant unmet needs remain in the management of this cancer. Here, I review the history of targeted therapies in neuroblastoma, focused on treatments that progressed to clinical evaluation and those expected to be impactful clinically in the near-term.

## 1. Introduction

Neuroblastomas are embryonal tumors arising from the sympathoadrenal precursors of the sympathetic nervous system, with the overwhelming majority of tumor arising from the adrenal glands or the sympathetic chains running parallel to the spine. While such tumors were described in the late 1800s, Wright first described the embryonal neural histopathology of the tumors and coined the term neuroblastoma [1]. Most cases of neuroblastoma at the time, like most cancers, were lethal, though the first successful surgical resection was reported in 1917 [2]. Radiation therapy was reported as early as 1928 [3], and multimodal therapy including radiation, chemotherapy and surgery by 1953 [4]. Advances were made for low-risk and intermediate-risk malignancies over that time, particularly with the development of histopathologic classification, anatomic staging and clinical risk stratification [5,6]. However, outcomes for patients with high-risk disease languished until the development of protocols using high-dose chemotherapy with autologous stem cell rescue [7,8,9]. While these approaches did significantly improve disease-related survival by 15–20%, they came with significant toxicity and long-term impacts on growth, development, and quality of life. With this history, it had long been appreciated that biology-driven targeted therapies were necessary to further improve outcomes in children with neuroblastoma while mitigating longitudinal harm.

**Figure 1:**
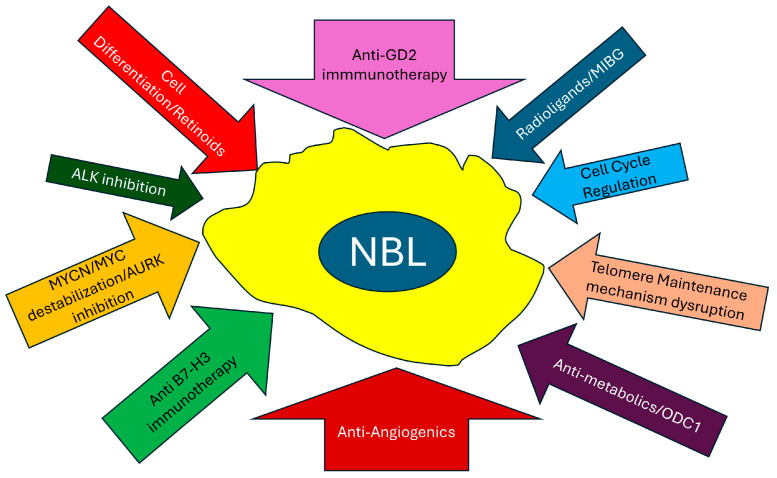
Summary of pathways evaluated for therapeutic impact in clinical trials for neuroblastoma.

In this review, I will discuss the landscape of targeted therapies in neuroblastoma, summarized in Figure 1 by pathway. I will highlight prior successes, particularly the paradigm-shifting benefits of anti-GD2 therapies, and ongoing work, organized by therapeutic target and disease biology, focusing on therapies that have reached or are likely to soon reach clinical trials. I will also discuss future directions and opportunities for these patients.

## 2. Cell Differentiation and Retinoids

Neuroblastoma is an embryonal tumor with an aberrant premature halt in differentiation. As such, researchers examined mechanisms by which to induce differentiation or apoptosis. Retinoids, derivatives of vitamin A, had been studied as early as the 1970s and shown to have effects on numerous malignant cells [10], including neuroblastoma [11,12,13]. All-trans retinoic acid had efficacy in vitro, but it was determined that 13-cis retinoic acid was more efficacious in vivo [14] due to improved stability outside of cells and autoisomerization intracellularly to the all-trans configuration. It is worth noting that early studies did demonstrate differential effects of either drug on neuroblastoma cells, with sensitive cells undergoing terminal differentiation while resistant cells would have a transient growth pause followed by proliferation after removal of the drug [15,16,17]. Nonetheless, this work led to inclusion of 13-cis retinoic acid in pediatric clinical trials. Initial studies failed to show benefit in monotherapy [18], but it was subsequently trialed in regimens after consolidation with high-dose chemotherapy with autologous stem cell rescue. In the British clinical trial, the addition of 13-cis retinoic acid did not improve event-free survival [19]; in contrast, the Children’s Cancer Group study comparing both use of high-dose chemotherapy and use of 13-cis retinoic acid demonstrated greatest benefit in patients who received both therapies [20]. This has led to the inclusion of 13-cis retinoic acid as a standard-of-care maintenance therapy in the US.

Additional efforts have been made to further refine retinoids for greater potency. Of these agents, fenretinide [21,22] has most significantly progressed to early-phase clinical trials. The Phase 2 study of single-agent fenretinide operated through the Children’s Oncology Group (COG) demonstrated one partial response and 13 patients with prolonged stable disease, with low oral bioavailability considered to limit activity [22]. Multiple approaches to further improve efficacy and potentially overcome resistance have been pursued, including micellar formulations and combination use with other agents [23,24,25,26,27,28,29,30], though none have been efficacious enough for clinical use.

## 3. GD2 and Anti-GD2 Immunotherapies

GD2 is a disialoganglioside, a glycolipid, that is expressed on cells of neuroectodermal origin [31], including neuroblastoma cells, melanoma cells, and to a lesser degree neurons, melanocytes and peripheral nerve fibers. Aside from being a component of the cell membrane, it appears to have diverse functions, including being an immune checkpoint on T-cells [32]. The robust expression on neuroblastoma cells and downregulation on mature tissues made it an attractive therapeutic target, with monoclonal antibodies first developed against GD2 in the 1980s and 1990s [33,34]. The initial antibodies were fully mouse anti-human. These were effective in vitro, including for the use of purging of neuroblastoma cells from bone marrow intended for transplant purposes [35], but they were associated with high rates of neutralizing human anti-mouse antibodies and toxicities, particularly pain.

To avoid these complications, researchers modified the original antibody. One group elected to modify the entire antibody, replacing the mouse constant regions with a human heavy chain IgG1 and light kappa chain [36]. This design proceeded through clinical trials, attaining approval as dinutuximab in the US in 2015, generated in murine cells [37], and dinutuximab beta in Europe in 2017, generated in Chinese hamster ovary cells [38]. Dinutuximab achieved landmark success in the Children’s Oncology Group study ANBL0032 [39], in which patients were randomized to 13-cis retinoic acid with or without dinutuximab, interleukin-2 and granulocyte-macrophage colony stimulating factor. This study was halted early after survival analysis demonstrated an absolute 20% improvement in event-free survival at 2 years, with durable effects on survival at 5 years [40]. Similar outcomes were later demonstrated with dinutuximab beta, used alone or with interleukin-2 alone [41]. In both cases, while early survival improved, later relapses were eventually identified, with a 5-year event-free survival advantage of 15–20%.

In parallel, researchers at Memorial Sloan Kettering Cancer Center [42] and at St. Jude’s Children’s Research Hospital [43] made other modifications to the anti-GD2 antibody. In both cases, changes were made to the rest of the antibody structure, generated from human genes but retaining complementary sequences from the mouse antibody. These “humanized” antibodies were expected to be less immunogenic; additional changes were made to reduce reliance on complement-dependent cytotoxicity and reduce pain and off-target inflammation. The product from Memorial Sloan Kettering demonstrated efficacy in patients with relapsed or refractory neuroblastoma and received FDA approval as naxitamab in 2020 [44]. The humanized antibody from St. Jude’s was shown to be safe and efficacious for use in combination with chemotherapy in frontline treatment [45], though it continues through the process for FDA approval. Research also continues examining additional modifications of these antibodies, such as conjugation with interleukin-15 [46] or interleukin-21 [47], use of modified interleukin-2 [48] or chemotherapeutics such as exatecan [49], with some agents entering early-phase clinical trials.

Alternative approaches to targeting GD2 which utilize more direct cytotoxic effects have also been examined. The generation of chimeric antigen receptor (CAR) T-cell therapies against GD2 has been ongoing for over 20 years, with modifications of the constructs to optimize peak cell proliferation, activity, and viability [50,51,52,53,54]. Of these, a third-generation CAR-T cell yielded the greatest benefit reported to-date, with a 63% overall response rate in a Phase 1/2 study with manageable safety and tolerability [55], though it is noteworthy that a significant number of the patients on that trial had minimal disease burden at enrollment. Nevertheless, despite the optimization of the CAR-T cells, prediction of benefit (i.e., patient factors of sensitivity) remains challenging. Other cell therapeutics, including CAR-NK cells [56,57] and T-cell receptor engineered cells [58,59], continue to be developed preclinically. GD2 has also been studied as a target for radiopharmaceuticals. Of these, the self-assembly disassembly pre-targeted radiotherapy (SADA-PRIT) platform [60], which first binds the therapeutic target with its proprietary unlabeled tetramers then acquires radioactive payloads, continues in early-phase clinical trials. Additional novel molecules, including bispecific antibodies [52,61], are in development but remain in preliminary stages.

With these efforts, researchers have recognized that the immunosuppressive tumor microenvironment (TME) significantly impairs the efficacy of anti-GD2 mAbs in a significant proportion of patients. Preclinical data support this hypothesis, as studies targeting in the TME including CD47/SIRPα [62,63] and CD105 [64] result in impaired tumor growth and augmented efficacy of anti-GD2 immunotherapy. Clinical translation of this work continues but has been challenging due to the on-target off-tumor adverse effects of therapeutics against these markers, particularly effects on the bone marrow and resultant cytopenias. Nonetheless, such mechanisms will be necessary to improve all immunotherapies, including those against GD2, in neuroblastoma.

## 4. Norepinephrine Transporter (NET), Somatostatin Receptors and Radiopharmaceuticals

Neuroblastomas arise from sympathoadrenal precursors and retain many of the biological features of these neuronal cells. As sympathetic cells function by secretion and reuptake of epinephrine and norepinephrine, >95% of neuroblastomas retain these functions, including expression of the norepinephrine transporter (NET). Clinicians have leveraged the expression of this transporter as a diagnostic and therapeutic vulnerability. Metaiodobenzylguanine (MIBG) is a synthetic analog of norepinephrine, originally created to function as an imaging agent of the adrenal medulla; it was instead found to be very specific for neuroendocrine tumors, including neuroblastoma [65]. MIBG was repurposed; when made with I^123^, it can serve as a diagnostic nuclear pharmaceutical [66,67], with emission detectable by single-photon emission computed tomography (SPECT); its short half-life allows for rapid clearance after imaging.

When MIBG is synthesized with I^131^, in contrast, it can be infused as a therapeutic radiopharmaceutical. The process requires multiple steps including patient isolation, specialized infrastructure and potential use of procedural sedation [68]. A significant proportion of patients may have subsequent cytopenias, so most protocols include a subsequent infusion of the patient’s own, previously collected and stored, peripheral blood stem cells [69,70]. Patients then recover over the subsequent 6–8 weeks. Its effects appear to be two-fold. There is an initial direct cytotoxicity from the radioactivity at sites of uptake, which is dependent on drug penetrance into sites of disease and overall disease bulk, with particularly bulky disease and micrometastatic disease comparatively less affected due to inadequate uptake. There is a subsequent immunologic response, likely due to the presentation of tumor specific antigens with the first wave of cytotoxicity; this results in ongoing antineoplastic activity observed over many weeks. The blood stem cell infusion is theorized to augment this immune response.

Therapeutic MIBG has been studied in multiple contexts with neuroblastoma. In the relapsed/refractory disease setting, it has objective responses rates between 14 and 36%, though clinical benefits rates are generally >75%, particularly with improvement in cancer-related pain [71,72,73,74]. It does work best in patients with either bone/bone marrow disease only or soft tissue metastases only and is less efficacious in patients with both types of metastases. A Phase 2 study showed potential benefit in the addition of MIBG therapy to high-dose chemotherapy with autologous stem cell rescue [75]; this result has been evaluated in the Children’s Oncology Group Phase 3 clinical trial ANBL1531, though results are pending. Multiple studies have evaluated the addition of different antineoplastic agents with MIBG [71,72,76,77,78]. Prior studies also demonstrated that serial infusions of MIBG generally demonstrated little additional benefit after the first infusion [79], likely due to the downregulation of expression of NET in the surviving tumor. Nonetheless, it remains a useful palliative therapeutic and may still have a role within induction and re-induction therapy.

The Somatostatin Receptor (SSR) is another protein consistently found to be expressed in neuroblastomas [80], though at a lower rate than NET. A different diagnostic and therapeutic radiopharmaceutical pair, Gallium Ga68 DOTA-TATE and Lutathera (lutetium Lu177 DOTA-TATE), has been developed against SSR for use against Somatostatin Receptor-positive gastroenteropancreatic neuroendocrine tumors, receiving FDA approval for use in patients 12 and older. Unfortunately, a clinical trial in patients with relapsed/refractory neuroblastoma demonstrated no significant benefit from Lutathera, with multiple potential reasons theorized by the researchers, including heterogenous SSR expression, inadequate total retained dose, and an inefficacious dosing schedule [81]. New radiopharmaceuticals are in development using alpha-decaying radioisotopes; these drugs have far less toxicity due to the limited penetrance of the radioactivity into adjacent tissues, which allows for the administration of a considerably higher dose. These radiopharmaceuticals are currently being evaluated preclinically for neuroblastoma [82,83,84,85,86].

## 5. ALK

The ALK gene is part of the MAPK signaling pathway, which has been long recognized to promote cell proliferation and viability. Aberrations in ALK, either as hotspot gain-of-function mutations or less commonly whole gene amplifications, are identified in ~10–20% of high-risk neuroblastoma tumors at diagnosis as a clonal or subclonal population [87,88]. These rates increase at relapse, due to either selection of subclonal populations identified within the original tumor or de novo mutations identified at relapse [89,90]. Most of these mutations are somatic and restricted to the tumor, although a small percentage of patients harbor germline mutations and are predisposed to neuroblastomas [91]. In any of these situations, the ALK mutation contributes to disease biology and is therapeutically targetable. There are a number of ALK inhibitors that are approved for use in adults with non-small cell lung cancer. Two have been evaluated in clinical trials thus far in children with neuroblastoma, crizotinib and lorlatinib. Crizotinib was determined to be safe in children, though with some potential of thrombosis; a recommended Phase 2 dose was identified, but this was less efficacious in neuroblastoma as compared to anaplastic large cell lymphoma [92]. Lorlatinib, in contrast, was demonstrated to have efficacy in an early-phase trial in ALK-mutated neuroblastoma specifically [93]; a recommended Phase 2 dose is now being studied in combination with chemoimmunotherapy in an ongoing Phase 3 clinical trial in children with ALK-aberrant neuroblastoma.

There has been new data that suggests that ALK may have a novel function in neuroblastoma. Wild-type ALK is highly expressed on most neuroblastomas, but direct inhibition with ALK inhibitors has no meaningful benefit. In preclinical models, the extracellular domain of the ALK protein is cleaved commonly; this is associated with increased cell migration and metastatic potential [94]. Inhibition of this cleavage results in decreased dissemination. Ongoing work will evaluate if this inhibition of cleavage is translatable to the clinic setting, which would offer a new therapeutic approach for all patients with neuroblastoma.

## 6. Aurora Kinases, MYCN and MYC

MYCN and, to a lesser degree, MYC have both been well-demonstrated to be strong oncogenic drivers of neuroblastoma, with pluripotent effects on proliferation, metastasis, chemoresistance and immortalization through telomerase activation. However, multiple aspects of these transcription factors make them challenging to target directly. These include the lack of catalytic domains to functionally inhibit intrinsically disordered structures limiting binding by drugs or antibodies, and pluripotent effects that can result in on-target, off-tissue toxicities with inhibition. Instead, researchers have examined indirect approaches to target MYC/MYCN biology. This work identified Aurora Kinase A (AURKA) as a candidate. This protein has multiple functions in neuroblastoma cells, including stabilization of mitotic spindle necessary for mitosis and stabilization of MYCN and MYC [95]. Preclinical studies demonstrated that inhibition of the kinase function of AURKA impaired its functions and destabilized MYCN/MYC, inducing apoptosis in single-agent use in vitro and in animal models of the disease [96,97,98]. The first AURKA inhibitor to reach clinical trials, alisertib, unfortunately had undue toxicity, particularly myelosuppression, preventing sufficient dosage that could induce cytotoxicity to neuroblastoma cells [99]. The drug was initially deprioritized by its manufacturer, though recently it has been relicensed and is being evaluated again, albeit in carcinomas. In our prior studies, we were able to demonstrate that combined inhibition of bromodomain and extraterminal (BET) proteins, epigenetic modifiers that upregulate the expression of MYCN/MYC, and AURKA could result in synergy, allowing for sufficient cytotoxicity at lower doses of each drug. This approach increased cell death in vitro and, in combination with vincristine, could induce durable complete tumor regression in cell line xenografts in SCID mice [100]. Such approaches could identify a role for AURKA inhibition in neuroblastoma.

AURKB, a paralog aurora kinase which is upregulated by MYCN and has additional functions in mitosis, was identified as another potential target [101]. Kinase inhibitors that directly targeted AURKB were evaluated preclinically and, while promising, were not developed further for clinical use. New agents continue in development, including multikinase inhibitors designed to inhibit AURKA and AURKB [102,103].

## 7. Telomere Maintenance Mechanisms, ATR, and ATM

Over time, studies into neuroblastoma have identified dynamic heterogeneity of cell populations, both of the neuroblasts themselves and of recruited cells (discussed further below). Among the neuroblasts, distinct populations of proliferative and quiescent cells were defined, the former representing a bulk population of the tumor mass and the latter as a more basal population, able to generate new proliferative populations as needed but maintain tumor viability overall. These cells remained viable through multiple mechanisms including the maintenance of telomeres, a key feature of cell immortalization. These telomere maintenance mechanisms (TMMs) include overexpression of the telomere genes driven directly by MYCN [104], gene rearrangements resulting in constitutively active promoter regions of these genes [105], or by alternative lengthening of telomeres (ALTs) [106], a “brute force” approach that effectively recruiting fragmented DNA to be attached at chromosome ends, serving as proxy telomeres. Regardless of the mechanism, patients whose tumors had activated TMMs were shown in multiple cohorts to have considerably worse response to treatment and overall survival [105,107,108,109,110]. This identified these mechanisms as prime candidates for new therapies.

How to target these mechanisms has been considerably more complicated. A perhaps obvious approach would be to directly inhibit the telomerase machinery itself, as it is a complex made of individual enzymatic components, scaffolding proteins, and template RNAs. One such direct telomerase inhibitor, imetelstat, has been developed, with preclinical evidence of efficacy, alone or in combination with the antimetabolite 6-thio-deoxyguanine. However, the Phase 1 clinical trial in children using the drug alone showed significant myelosuppression and low rates of responses [111]; the drug was deprioritized for development for pediatric solid tumors. There are ongoing preclinical efforts to directly and specifically inhibit telomerase in neuroblastoma while sparing other proliferative cells, but these remain in very early stages of development.

Parallel to those efforts, researchers have been examining other components of TMM that could be targetable, particularly in a more tumor-specific fashion. The proteins ATR and ATM, serine threonine kinases with key functions in DNA Damage Response (DDR), have been demonstrated preclinically to have key functions within both conventional telomerase activity as well as ALT [112,113,114]. The mechanisms by which these proteins promote or regulate TMM are still being elucidated, but preclinical studies demonstrated that inhibitors of either protein can impair cell proliferation and viability in vitro and in vivo. Among these inhibitors, elimusertib, an ATR inhibitor, has advanced to clinical trials including a pediatric Phase 1/2 trial through COG (NCT05071209). That study has identified a safe and tolerable dose for children with relapsed/refractory pediatric solid tumors; however, elimusertib has been deprioritized for development in children. Nonetheless, other ATR and ATM inhibitors remain in early-phase development, holding some promise of development for neuroblastoma.

## 8. Cell Cycle Kinetics and Cyclin Regulation

Neuroblastoma cells manipulate the kinetics of the cell cycle to maintain viability, as reviewed here [115]. The cells must regulate DNA damage repair pathways to allow for the accumulation of genomic alterations that drive oncogenesis, metastasis and therapy resistance. However, neuroblastomas must also maintain cell division through cell cycle checkpoint kinase expression and phosphorylation to permit sufficient tumor growth and viability. These unique functions have offered potentially tumor-specific therapeutic vulnerabilities for development.

Preclinical work first evaluated CDK4/6 as potential therapeutic targets, leveraging the development of inhibitors against those cyclin proteins that had been developed for breast carcinoma. Palbociclib [116,117], ribociclib [118,119] and abemaciclib [119,120] all demonstrated antitumor efficacy in slowing proliferation and inducing terminal differentiation in vitro and in animal models of disease. However, in clinical trials, benefits were more modest; single-agent trials showed some partial responses or extended stable disease [121,122], though one trial with palbociclib and chemotherapy is pending completion (NCT03709680). Other cyclins, including CDK2 [123,124], CDK7 [125,126] and CDK9 [127,128], have also shown potential preclinical benefit, with lead compounds inducing terminal differentiation with greater sensitivity than CDK4/6 inhibitors, but researchers are awaiting compounds to be advanced for pediatric trials.

In contrast, CHK1, WEE1 and CHK2 have been identified as other candidate targets [129,130,131,132]. These proteins, when activated by DNA damage, cause a cascade effect that results in cell cycle arrest, preventing rapid and unregulated mitosis, including through the inhibition of cyclins including CDK2. Inhibitors of these three proteins prevent cell cycle arrest and induce mitosis, including through the activation of CDK2; this dysregulation, somewhat paradoxically, destabilizes neuroblastoma cells and can lead to apoptosis and tumor death. The first WEE1 inhibitor evaluated in pediatric clinical trials, adavosertib, showed excessive hematologic toxicity with carboplatin but was comparatively well-tolerated with irinotecan [133,134]. A modest number of patients had responses across two clinical trials, though it is worth noting that patients with ALT-activated tumors had durable responses. New classes of agents, such as molecular degraders or glues, may be more beneficial with narrower antitumor-specific profiles; combinations with other agents may also take advantage of synthetic lethality in a more targeted way than conventional chemotherapies can offer.

## 9. Antiangiogenic Therapies

Angiogenesis has long been appreciated to be a poor prognostic feature of neuroblastoma, associated with metastatic dissemination and poor survival [135]. Numerous biomarkers were subsequently identified to correlate with angiogenesis, including IL8/IL8R [136], integrins [137,138], and VEGF/PDGF/VEGFR pathways [139]. Antiangiogenic therapies have been subsequently tested against preclinical models of disease. These studies would demonstrate promising activity [138,140,141,142,143,144,145], but translation to the clinical setting was challenging due to toxicity and diminished efficacy. In addition to the direct antiangiogenic drugs and tyrosine kinase inhibitors tested above, studies demonstrated that chemotherapy [146,147] and fenretinide [148] also natively have antiangiogenic activity. This sustained interest in additional studies.

Direct VEGF inhibition with bevacizumab has been shown in multiple preclinical models to be efficacious against neuroblastoma [149,150] and in a patient for whom it was used under compassionate use [151]. Parallel studies demonstrated that bevacizumab could be used safely in children [152,153], allaying concerns of impact on growth and development. These studies have culminated in the BEACON-Neuroblastoma protocol through the SIOPEN (International Society of Paediatric Oncology Europe—Neuroblastoma) collaborative group. In this study, bevacizumab improved overall response rate in combination with irinotecan and temozolomide, meeting criteria for further study [154]. Integration into the next series of clinical trials remains under consideration.

Multitargeted tyrosine kinase inhibitors (TKIs) have also been evaluated in neuroblastoma as antiangiogenic therapies. A mixed blessing of these drugs is their promiscuity; they can target multiple angiogenic pathways including VEGFR, TEK, and AXL and other oncogenic pathways, depending on the specific TKI of interest. This can result in more pluripotent effects but also in increased toxicities. In preclinical models, many TKIs have shown antitumor efficacy, through antiangiogenic and cytostatic effects [155,156,157,158,159,160,161,162,163]. However, in the absence of a target driver mutation, most of these TKIs can be expected to have at best a cytostatic effect. Nonetheless, we hypothesized that cabozantinib could function to delay or prevent tumor recurrence in patients after prior treatment. We reported that single-agent use of cabozantinib in four children with relapsed neuroblastoma resulted in sustained disease control [164]. Based on these data, in the CaboMain trial (NCT05135975) we are evaluating the efficacy and tolerability of one year of cabozantinib in patients with “ultra-high” risk disease, including those with relapsed neuroblastoma who achieve stable disease or better after reinduction therapy. This study is ongoing.

Additional research discovered that the combined use of the mTOR inhibitor sirolimus, also known as rapamycin, synergized with chemotherapy for antiangiogenic effects against preclinical models of neuroblastoma [147] with subsequent cell cycle arrest and tumor regression. A subsequent study of vinblastine and rapamycin in patients with relapsed neuroblastoma identified a safe and tolerable dosing regimen with one patient with a partial response and three with prolonged stable disease. These results along with the data on TKIs led to the consideration of the multidrug combination of rapamycin, irinotecan, dasatinib and temozolomide (RIST) for neuroblastoma. In a Phase 2 trial, RIST showed significant efficacy in patients with MYCN-amplified disease in prolonged disease-free survival, with a numerical but statistically nonsignificant improvement in patients with MYCN-nonamplified disease [165]. This approach is considered a reasonable approach for patients with relapsed disease [166], particularly those who have progressed on anti-GD2 therapies, and it merits further study.

## 10. B7-H3

CD276, also known as B7-H3, is a member of the B7 family of immune checkpoint proteins and is highly expressed on a number of malignant cells, including neuroblastoma [167]. Interestingly, it seems to be rarely expressed on the surface of normal tissues, making it highly attractive as a “pan-cancer” marker both as a simple tumor-restricted surface antigen as well as for inhibition to reactivate the patient’s anticancer immunity.

For patients with neuroblastoma, the first therapeutic against B7-H3 was an IgG1 monoclonal antibody that was first characterized by its specificity for tumors [168]. Designated at 8H9, its target was later characterized to be B7-H3 [169]. This antibody was optimized and modified over time, developed clinically as omburtamab. Researchers further optimized the antibody through conjugation of cytotoxic agents, including initially cobra venom toxin and eventually the radioisotope ^131^I [170]. This molecule was found to be rapidly bound in the liver, limiting its use for systemic therapy; however, it was evaluated for delivery into the cerebrospinal fluid (CSF) space for patients with neuroblastoma with leptomeningeal or CNS disease, as well as for other pediatric CNS tumors [171]. Initial studies showed promise, in combination with systemic chemotherapy and external beam radiation therapy [170,172]. In the Phase 2 study (NCT03275402), patients with CNS metastases of neuroblastoma showed response to omburtamab. However, the study was deemed to be not supportive of FDA or EMA approval due to the control group not receiving comparable therapy, particularly external beam radiation therapy [173]. Further development remains unclear.

In parallel, multiple efforts have been made to target B7-H3, particularly for systemic delivery. At present, antibody drug conjugates against B7-H3 have been developed by academic groups and pharmaceutical companies, of which at least one, Ifinatamab deruxtecan [174], demonstrated to have preclinical in vivo efficacy against neuroblastoma and other pediatric solid tumors. Other researchers are optimizing chimeric antigen receptor (CAR)-engineered cells, including T-cells [175] and NK [176] cells, against B7-H3. Clinical trials of these agents against neuroblastoma are expected in the next few years.

## 11. ODC1 and Metabolomics

An area of active research across oncology has been the role of metabolomics. One of the “Hallmarks of Cancer,” alternative mechanisms of energy utilization and catabolic byproducts offer cancer-specific pathways that can be targeted with less impact on otherwise healthy tissues. In neuroblastoma, particularly those driven by MYCN/MYC biology, ODC1, the gene that encodes ornithine decarboxylase is transcriptionally upregulated, resulting in a reliance on the ODC cycle as an alternative pathway to energy production as opposed to the Kreb cycle. Researchers identified that the antiparasitic agent difluoromethylornithine (DFMO), also known as eflornithine, can inhibit the function of ODC1 and induce a metabolic crisis [177]. Interestingly, DFMO had been studied as early as the 1980s in neuroblastoma [11], in parallel with isotretinoin [178]; some preclinical studies even suggested there could be some combinatorial effect, if not outright synergy, in using the two drugs together [179].

Regardless, preclinical studies found that MYCN-amplified tumors were sensitive to eflornithine, particularly at higher concentrations of exposure, impairing tumor growth and new tumorigenesis [180,181,182]. Neuroblastoma cells without MYCN amplification but with MYC overexpression similarly showed sensitivity to DFMO. Based on these results, there were two different efforts to translate DFMO to the clinic. One approach was single-agent DFMO, used at the completion of frontline treatment of high-risk neuroblastoma, including anti-GD2 immunotherapy, in patients who had achieved a complete response prior to post-consolidation therapy. The dose selected for use was arbitrarily decided to be 500 mg/m^2^/day divided twice daily, for a four week cycle, for up to 27 cycles. Other nuances included that patients and their caregivers were given a list of food with high, moderate, or low levels of polyamines, the nutritive compounds that are depleted in the course of treatment with DFMO; while not explicitly told to avoid these foods, it can be presumed that some families reduced intake of those foods, with unknown impact on efficacy. A retrospective propensity score matching approach was taken to compare the experimental cohort to the historical control, using patients from the prior COG high-risk neuroblastoma clinical trial. In that analysis, it was found that patients who received DMFO had a 96% 4-year overall survival, as compared to 84% in the matched cohort [183]. However, the pharmacokinetics of the drug in vivo were not high enough to affect ODC1 and impair cell metabolomics, as based on the foundational preclinical work that justified the clinical trial. Nonetheless, based on the clinical trial results and the generally low rates of toxicity, DFMO was approved by the FDA, albeit without a unanimous decision at the FDA ODAC session, for use as a maintenance therapy after completion of frontline therapy [184].

A parallel effort, through collaboration of multiple laboratories, evaluated the efficacy of high-dose DFMO. At doses of 2500 mg/m^2^/day, plasma concentrations of DFMO do rise to levels that are inhibitory of ODC1 [185,186]. This dose was used in the COG clinical trial ANBL1821 in combination with chemoimmunotherapy. The initial Phase 2 trial (NCT03794349) had to be halted temporarily, unfortunately, at this dose because of unexpectedly higher rates of Grade 3 hearing loss, a known potential toxicity of DFMO but considered to be potentially reversible. After a modification of the dosing approach, the clinical trial was completed; use of DFMO did not significantly improve response rates or progression-free survival as compared to chemoimmunotherapy [187]. Interestingly, this higher dose of DFMO was used in an additional stratum of the clinical trial using it in single-agent use at end of frontline therapy, and it was considerably more toxic when used daily, with significant hearing loss and GI toxicity, requiring multiple dose reductions in most patients to be tolerated; this study is ongoing (NCT02679144). Continuous “low-dose” DFMO has also been tested with various combinations of chemotherapy in the relapsed/refractory disease setting, with final results pending but initial results not dissimilar to the results from ANBL1821 [188]. Further studies are elucidating other potential biological functions of DFMO; for example, researchers of gliomas have identified roles of ODC1 and polyamines on tumor microenvironment and immune tolerance of cancer cells [189,190] or modulation of ongoing mutagenesis [191]. Studies on the functions in neuroblastoma may indicate novel treatment combinations for further study.

## 12. Conclusions

The summary above is a focused review of the efforts into improved outcomes for patients with high-risk neuroblastoma through targeted therapies, with critical trials encapsulated in Table 1. Although the aggressive multimodal approaches used to care for these patients have improved five-year overall survival to ~60% in much of the developed world, only the post-consolidation approaches, including anti-GD2 immunotherapy, retinoic-acid-induced differentiation, and the use of eflornithine (through a cryptic mechanism of action), have been truly “targeted,” focusing specifically on neuroblastoma biology. Future improvements in care are expected through focus on immunotherapeutic approaches, including antibody drug conjugates, bispecific antibodies, and cell therapies. Other therapeutics with novel mechanisms of action, such as proteolysis targeting chimeras (PROTACS) [103,192] and molecular “glue” degraders [193], may further augment on-tumor efficacy with reduced off-tumor toxicity. Biological approaches must address MYC/MYCN biology, telomere maintenance mechanisms, and the permissive tumor microenvironment which shields tumors from antineoplastic activity; metabolomics and cell cycle dependencies may be additionally leveraged as novel targets. A combination of these approaches must be used to optimize neuroblastoma therapy, leading to more nuanced therapies with less general toxicity and improved overall outcomes.

## Figures and Tables

**Table 1:** Summary of key clinical trials with outcomes supporting treatment use.

Study Publication	Key Study Drug and Relevant Outcome Measures	Number of Study Participants	Results
Matthay et al., 2009 [20]	13-cis retinoic acid (RA)and myeloablative chemotherapy with autologous stem cell rescue (autoBMT) as consolidation after frontline induction chemotherapy; 5-year overall survival	397 (two randomizations, 1:1 for autoBMT; 1:1 for RA)	5-year overall survival: Chemotherapy alone, 36% ± 7%; Chemotherapy with RA, 38% ± 7%; Chemotherapy with autoBMT, 41% ± 8%; Chemotherapy with autoBMT and RA, 59% ± 8% *p* < 0.01
Yu et al., 2010 [39]; Yu et al., 2021 [40]	RA with or without Dinutuximab with alternating cycles of IL2 or GM-CSF as post-consolidation of frontline therapy; 2-year and 5-year event-free survival and overall survival	226 (randomized 1:1)	2 year measures: RA alone: EFS 46% ± 5%; OS 75% ± 5% With dinutuximab: EFS 66.5% ± 5%; OS 86% ± 4% 5-year measures: RA alone: EFS 46.1% ± 5.1%; OS 56.6% ± 5.1% With dinutuximab: EFS 56.6% ± 4.7%; OS 73.2% ± 4.2%
Ladenstein et al., 2018 [41]	Dinutuximab beta without or with subcutaneous IL2 as post-consolidation of frontline therapy; 3-year event-free survival	406 (200 dinutuximab beta alone; 206 with IL2)	3-year EFS: Dinutuximab beta alone: 56% (95% CI 49–63% With IL2: 60% (95% CI 53–66%)
Mora et al., 2025 [44]	Naxitamab with GM-CSF (single arm); overall response rate in relapsed/refractory disease	74 (50% relapsed, 50% primary refractory)	ORR: 50% (95% CI 36–64%); 58% of responders with primary refractory disease, 42% with relapsed disease; one year overall survival 93% (95% CI 80–98%); one year progression-free survival 35% (95% CI 16–54%)
Locatelli et al., 2025 [55]	GD-2 targeted autologous CAR-T cell (single arm); safety, maximally tolerated dose, overall response rate and complete remission rates in patients with relapsed disease	35 within primary trial	No new safety signals; four children with ICANS requiring cell inactivation; MTD 10 × 10^6^ cells/kg; ORR 66% of patients with disease; CR 40% at 6 months; CAR-T cell persistence at 12 months 64%; 5-year overall survival 42.67%
Yanik et al., 2015 [75]	I^131^-MIBG therapy with high-dose chemotherapy with autologous stem cell rescue (single arm) and external beam radiation; response rate in primary cohort	50; 27 with primary refractory disease, 15 with progressive disease, 8 in secondary cohort with partial response only to frontline induction therapy	ORR 10% of primary cohort, 38% of secondary cohort; 3-year event-free survival in primary cohort 20% ± 7%; 3-year overall survival in primary cohort 62% ± 8%
Goldsmith et al., 2023 [93]	Lorlatinib with or without cyclophosphamide and topotecan (CyTopo); safety, pharmacokinetics and recommended Phase 2 dose (RP2D) in patients with relapsed or refractory disease	25 ages 1–17 years lorlatinib alone; 15 18-years or older lorlatinib alone; 9 with lorlatinib and CyTopo	RP2D 115 mg/m^2^/day in children, with or without CyTopo; RP2D 150 mg daily in adults; single-agent ORR 30% < 18 years, 67% ≥ 18 years; lorlatinib with CyTopo ORR 63%
Moreno et al., 2024 [154]	Temozolomide with or without irinotecan or topotecan, with or without bevacizumab in patients with relapsed or refractory disease; overall response rate	160; 3 × 2 factorial design, with 1:1 randomization to with or without bevacizumab	ORR: 18% (95% CI 10–28) without bevacizumab, 26% (95% CI 17–37%) with bevacizumab, *p* = 0.17; met protocol-defined ORR 1-year PFS bevacizumab-irinotecan-temozolomide 0.67 (95% CI 0.47–0.8)
Corbacioglu et al., 2024 [165]	Irinotecan and temozolomide alone or with dasatinib and rapamycin; progression-free survival in patients with relapsed/refractory disease	123 (66 in control group; 63 in experimental)	Median PFS in control group: 5 months (95% CI 2–8 months); in experimental group: 11 months (95% CI 7–17 months), *p* = 0.019; patients with MYCN-amplified disease, mPFS 2 month vs. 6 months, *p* = 0.012; patients with MYCN-nonamplified disease, mPFS 8 months vs. 14 months, *p* = 0.49
Oesterheld et al., 2024 [183]	Eflornithine after frontline therapy with partial remission or better (single arm), with post hoc external control propensity-score match survival analysis; 4-year event-free and overall survival	90 in primary analysis cohort (270 matched patients identified in external control, for 3:1 control/experimental ratio)	4-year EFS: control 72% ± 2%, eflornithine 84% ± 4% 4-year OS: control 84% ± 1%, eflornithine 96% ± 2%

## Data Availability

There are no original data that were developed or generated in the process of the writing of this manuscript. All cited work has indicated data availability statements.
